# Measuring the Impact of Environment on the Health of Large Cities

**DOI:** 10.3390/ijerph15061216

**Published:** 2018-06-09

**Authors:** Christine Stauber, Ellis A. Adams, Richard Rothenberg, Dajun Dai, Ruiyan Luo, Scott R. Weaver, Amit Prasad, Megumi Kano, John Heath

**Affiliations:** 1 School of Public Health, Georgia State University, Atlanta, GA 30303, USA; rrothenberg@gsu.edu (R.R.); rluo@gsu.edu (R.L.); srweaver@gsu.edu (S.R.W.); jheath1@gsu.edu (J.H.); 2 Department of Geosciences, College of Arts and Sciences, Georgia State University, Atlanta, GA 30303, USA; eadams23@gsu.edu (E.A.A.); ddai@gsu.edu (D.D.); 3 The Global Studies Institute, Georgia State University, Atlanta, GA 30303, USA; 4 Department of Information, Evidence and Research, World Health Organization, 1202 Geneva, Switzerland; prasada@who.int; 5 The World Health Organization Center for Health Development (The WHO Kobe Center), Kobe 651-0073, Japan; kanom@who.int

**Keywords:** urban health, health indicators, metrics, environmental indicators

## Abstract

The relative significance of indicators and determinants of health is important for local public health workers and planners. Of similar importance is a method for combining and evaluating such markers. We used a recently developed index, the Urban Health Index (UHI), to examine the impact of environmental variables on the overall health of cities. We used the UHI to rank 57 of the world’s largest cities (based on population size) in low- and middle-income countries. We examined nine variables in various combinations that were available from the Demographic and Health Surveys conducted in these countries. When arranged in ascending order, the distribution of UHIs follows the previously described pattern of gradual linear increase, with departures at each tail. The rank order of cities did not change materially with the omission of variables about women’s health knowledge or childhood vaccinations. Omission of environmental variables (a central water supply piped into homes, improved sanitation, and indoor solid fuel use) altered the rank order considerably. The data suggest that environmental indicators, measures of key household level risk to health, may play a vital role in the overall health of urban communities.

## 1. Introduction

There is growing evidence that environmental conditions will play an important role in global population health, especially in rapidly urbanizing low- and middle-income countries. Recently, the Lancet Commission on Pollution and Health estimated that pollution was responsible for 9 million premature deaths in 2015 alone, and that most of these (92%) occurred in low- and middle-income countries [1]. Furthermore, cities are severely impacted by pollution, as they occupy increasingly important roles in housing dense populations and in economic and industrial activity.

The health and well-being of urban dwellers result from the complex structures that shape their cities. Some urban conurbations are more successful than others in providing resources and a clean environment, but the specific mix of forces for success—environmental, economic, political, cultural, geographic, structural—is difficult to sort out. As an early step in the process of disentangling these elements, we examined the relationship of urban health, income, and income inequality to try to classify patterns of urban “success” in 57 cities across the world.

The literature on environment and health is voluminous, in part because both elements are multifaceted (a search for “built environment” and health in a scholarly database provides over 27,000 references). Due to the complex nature and interrelatedness of these elements, Knol et al. [2] reviewed and characterized the various conceptual frameworks for tackling complex environmental health problems. As an organizing principle, they proposed a three-level taxonomy that examines structural, relational, and operational approaches to examining and identifying solutions to major environmental health challenges [2]. This approach sorts out the structural framework, the interrelationship of elements, and operational models that can be of value for analysis and intervention [3]. One such relational framework, Driver–Pressure–State–Exposure–Effect–Action (DPSEEA), has been applied to the overall issue of water safety and offers an appreciation of the interrelation of its seven elements and the environment and links to human health [4]. Applying frameworks such as DPSEAA can enable users to support decision-making around key environmental areas of concern and identify areas for intervention [5].

Our specific interest in this study is a more modest application of the DPSEEA framework: to detect the influence of environmental indicators, specifically those associated with household-level risk (water access on premises, improved sanitation, and solid fuel use in the home) on assessments of the health and well-being of cities across the globe. Regardless of population size and level of income inequality, cities could significantly enhance health with improvements in water, sanitation, and hygiene (WASH) even under constrained resources. Luh and Bartram (2016) [6] recently analyzed progress on drinking water and sanitation in 73 countries and found that improvements in these services do not appear to be correlated with socioeconomic characteristics, but are more likely the result of policies and governance. Furthermore, cities all over the world are facing growing concerns of water stress in the face of population growth, rapid urbanization, and climate change [7]. Water demand is growing even as freshwater supplies are declining rapidly. Achieving improvements in health and well-being in cities thus calls for better tracking and understanding of the role of environmental indicators, especially in urban environments.

The tool that we use for these comparisons is the previously developed Urban Health Index (UHI) [8,9], a flexible composite metric that permits use of locally available data in combinations that are appropriate for local analysis. The UHI, when applied in local public health settings, provides information on key indicators and goals of interest for local or regional public health professionals. The tool is flexible enough for application in a variety of settings and enables users to set goals and targets as well as measure progress [9,10]. Recently, researchers used the UHI to analyze changes in geographic disparities and identify factors influencing those changes in major urban centers such as Atlanta and Rio de Janeiro [11,12]. The UHI can enhance decision-making by key stakeholders, target resources more effectively, or address underlying factors that worsen disparities in vulnerable populations. The UHI approach can also highlight outliers from the general trend and enable a better understanding of the variability in urban settings.

In an effort to better understand the health of cities, we selected the UHI approach to analyze data from the largest systematically collected database, the Demographic and Health Surveys (DHS) [3]. Although not designed for this purpose, data can be extracted and modified to examine the requisite variables in cities from the data available in the DHS. Under constraints of data availability, we examined different sets of urban markers and determinants, looking for a balance between the cogency of markers and their availability for the largest number of cities. To test the informativeness of different combinations of indicators, we compared sets of indicators with and without environmental indicators. The absolute values (health level) and relative values (comparisons with and without environmental markers) of the UHI were examined to develop an estimate of how environmental factors affect the overall well-being of a city. We compared several UHI constructs for the cities with the countries’ gross national income (GNI) and Gini Index, a standard measure of income inequality. These comparisons provide an estimate of how well cities can make use of available resources. Finally, we used two cities—Lagos, Nigeria, and Tegucigalpa, Honduras—to examine the intracity geographic distribution of the UHI.

## 2. Materials and Methods

### 2.1. Metrics

The Urban Health Index adopted methodology developed for the Human Development Index [13] to construct a multifactor single index for the level of health in a defined geographic area. In brief, the included indicators are standardized on a scale of 0 to 1 by dividing the indicator score minus the minimum score for the area by the maximum minus the minimum. The minimum may be the minimum observed for the grouping or an arbitrary minimum (such as approximately zero) to compare groupings. The indicators, all ordered in the same direction, are then combined by using their geometric mean to create the UHI for each unit. When the UHIs are rank-ordered and graphed (smallest to largest), they produce a distribution that typically diverges from linearity in the bottom and top 10% of UHIs. The ratio of the mean of the top 10% to the bottom 10%, referred to as the disparity ratio, can be interpreted as a relative risk. The slope of the middle 80% of the data is the disparity gradient. This slope can be interpreted as the extent of homogeneity within the grouping (a large positive slope suggests heterogeneity; a flatter line suggests greater homogeneity) [8]. A detailed document describing the approach along with an Excel spreadsheet to perform the calculations are available [9].

Gross national income (GNI) based on per capita purchasing power parity (PPP) was taken from the World Bank database [14]. Data on the Gini Index, a measure of inequality based on the simultaneous distribution of population and income, was also derived from World Bank data on member nations [15]. The Gini Index was available for only a subset of the major cities, so the country Gini Index was the primary marker.

### 2.2. City Selection

We derived indicators from all datasets available in the DHS data from 2003–2013. If multiple country datasets were available, we used the most recent survey. The DHS performs periodic country-wide surveys on a standard set of health-related issues using a multistage cluster sampling technique. The DHS is designed to be representative at the national level, at the urban or rural level, and at the regional level for departments or provinces/states. We selected the population capital city over the administrative capital city for this analysis. The cities with the largest population often have the largest numbers of survey respondents and therefore provide the largest numbers of observations for analysis. By selecting these cities, we were also able to explore intracity variability because of the high numbers of sampling clusters within the population capital cities.

Supplementary Table S1 provides details on the countries: year of data collection, selected city, household sample size, women’s and children’s sample sizes, and variables used to identify the city sample. The latter were required in order to identify the region of the country that was home to the capital city (or largest city). In some instances where exact designation was not possible, some combination of region and type of place was used, often with markers that were unique to a specific country. Additional selection details are provided in Appendix A. In all, 60 cities were identified within these datasets that had data available for most of the indicators proposed. Depending on the availability of indicators for determinants, between 43 and 60 cities were used in the analyses.

### 2.3. Indicator Selection

As noted, the indicator set was chosen based on the broadest availability and relevance to the study’s aims. Initially, there was interest in examining determinants of health that could provide proxy information on access to health care (such as access to prenatal care or treatment for diarrheal disease in children). However, these indicators only represent a smaller subset of the population in each location, as they are restricted to women who have been pregnant in the last 5 years or children under 5 years of age who experienced diarrheal disease in the 2 weeks preceding the survey. Therefore, a set of indicators that were social determinant–focused was selected, since it provided larger subsamples within each capital region, which can reduce the variability of the measures when compared across different cities. Another reason for selecting these determinant-focused indicators was that many were available in other datasets outside of the DHS, and they are closely linked with indicators from Sustainable Development Goals 3 and 6 [16].

The indicators, drawn from the household-level data, children’s data, and women’s data in the DHS, were grouped into the following domains: environment, education, infectious disease risk factors, chronic disease risk factors, and childhood immunization coverage (Supplementary Table S2). We explored different combinations of indicators to optimize the number and type of indicators and the number of included cities, and ended up with 9 that best fit the criteria. The final 9 indicators were selected based on inclusion in the DHS questionnaire during the 10-year period. To analyze the largest number of cities possible, the indicators associated with chronic disease risk (such as smoking and adult obesity) were not included, because they were missing from >20% of the countries selected. Each grouping (environmental, education, etc.) was selected for its relevance to global targets or goals such as the Millennium Development Goals (now Sustainable Development Goals) or the World Health Organization Global Health Observatory [17]. For example, the environmental indicators were selected because of their relevance to the Sustainable Development Goals for universal access to water and sanitation [16]. The variables associated with childhood immunizations were selected because they serve as proxy indicators for health care coverage [18]. Women’s health indicators regarding HIV were selected as a measure of behavioral risk factors.

These 9, designated UHI-9, consisted of *3 environmental indicators* (percent with water piped onto premises, percent with improved sanitation, percent without solid fuel used in the home); *3 indicators of women’s education and health* (percent of women who completed secondary education, percent of women who believed that having only 1 partner can decrease HIV risk, percent of women who believed that condom use can decrease HIV risk); and *3 indicators of childhood immunization coverage* (percent of children 1–4 years old who received 3 doses of diphtheria, pertussis, tetanus (DPT) vaccine, percent of children 1–4 years old who received 3 doses of polio vaccine, percent of children 1–4 who received at least 1 dose of measles vaccine). The values for these indicators are reported in Supplementary Table S3 for each included city. We were not successful at finding a single indicator set that was available for every city, and our final grouping of 9 indicators permitted analysis of 57 cities (in 54 countries).

### 2.4. Intracity Analysis

We identified 40 DHS clusters in Lagos, Nigeria, and 75 clusters in Tegucigalpa, Honduras. These cities were chosen to exemplify 2 contrasting world regions from which the most recent data were available (Nigeria in 2013, Honduras in 2011–2012) and provided sufficient numbers of clusters to apply the method. We used the UHI-9 for those areas to generate the cluster score maps. Geospatial placement of these clusters on their respective city maps was performed to demonstrate distribution of the UHI within the city.

### 2.5. Analytic Approach

We focused on the comparison of the UHI-9 with 3 versions of a UHI-6, each of which contained a different combination of 6 of the 9 variables: UHI-6A included environmental and women’s health variables, UHI-6B included women’s health variables and childhood vaccine coverage variables, and UHI-6C included environmental and vaccine coverage variables. We calculated descriptive variables for each of the UHIs (mean, median, standard deviation, minimum, maximum, disparity ratio, and disparity gradient), examined their correlations, and used the 4 UHI estimates to examine changes in the rank ordering of the cities. Graphs depicting change in rank with the removal of variable sets from the UHI-9 provided visual confirmation of the effect of including environmental indicators.

## 3. Results

### 3.1. Descriptive Statistics

**Figure 1:**
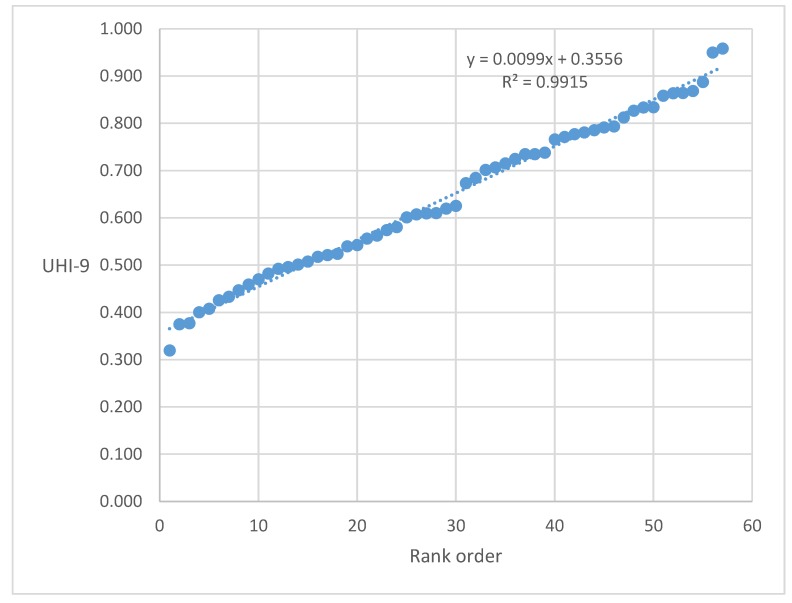
Scattergram of ordered Urban Health Index (UHI)-9 scores for 57 cities analyzed from Demographic and Health Surveys (DHS) data (2003–2013).

The nine-indicator UHI displayed typical features of previously observed groupings (Figure 1) [8,12,19]. The three other UHI-6 versions had similar configurations with somewhat different scales. Summary statistics for the four versions were generally similar (Table 1), but suggested some important differences. The highest UHI mean value was observed for version UHI-6B (women’s health plus vaccine coverage: 0.706). The addition of environmental variables (UHI-9) lowered the mean value to 0.642, indicating generally lower values for those variables. Similarly, the correlations of UHI-6B with UHI-9 (0.772), UHI-6A (0.716), and UHI-6C (0.618), all three of which contain environmental variables, are also suggestive of a disconnect in cities between personal health and environmental variables (as mentioned in Table 1, all correlation coefficients were statistically significant). Finally, UHI6-B had the lowest disparity ratio (1.677) and the mildest slope (0.0061), both indicative of greater uniformity among cities when only women’s health and vaccine variables were considered. The addition of environmental variables (UHI-9) increased the disparity ratio to 2.339 and the disparity gradient to 0.0099, indicating greater inequality in the grouping. The omission of childhood vaccine coverage (leaving women’s health and environmental variables) increased the disparity ratio even further, to 3.264, and the slope to 0.0122.

**Figure 2:**
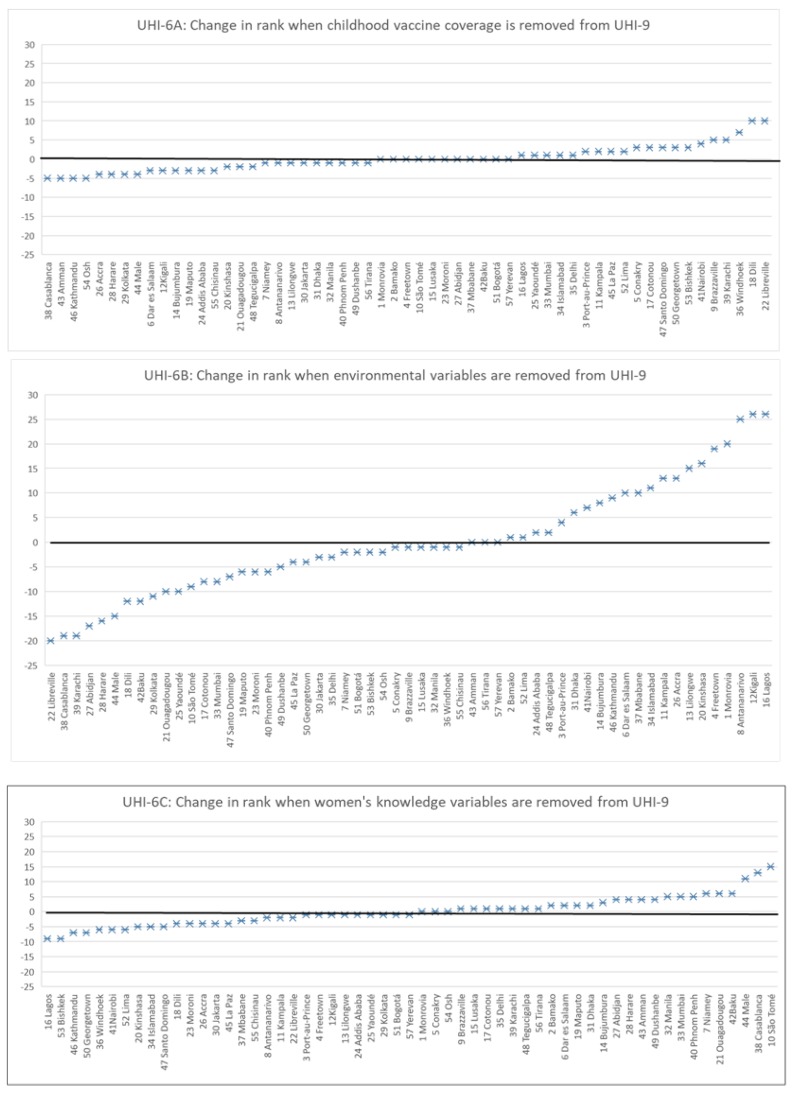
Comparison of changes in rankings of cities with different groupings of indicators.

The effects of different variable combinations are also demonstrated by examining the change in relative rank of cities with different indicator groupings (Figure 2). Removing childhood vaccine coverage from the UHI-9 (i.e., UHI-6A) alters the relative ranking to a small extent, with changes from −5 ranks (lower UHI ranking) to +10 ranks (higher UHI ranking) in a random pattern across the spectrum (Figure 2, upper panel). Similarly, when women’s knowledge variables are removed from UHI-9 (i.e., UHI-6C), the change is somewhat greater (from −9 to +15) but without a discernible pattern (Figure 2, bottom panel). When environmental variables are removed (UHI-6B), a major change occurs across the entire spectrum (from −20 to +26) (Figure 2, middle panel). Only nine cities remained at their previous positions. Cities with higher UHIs fell and those with lower UHIs rose in ranking. This suggests that adding environmental variables improves the overall assessment of health determinants for many cities and diminishes it for others.

### 3.2. Relationship of UHI to Income and Income Disparity

**Figure 3:**
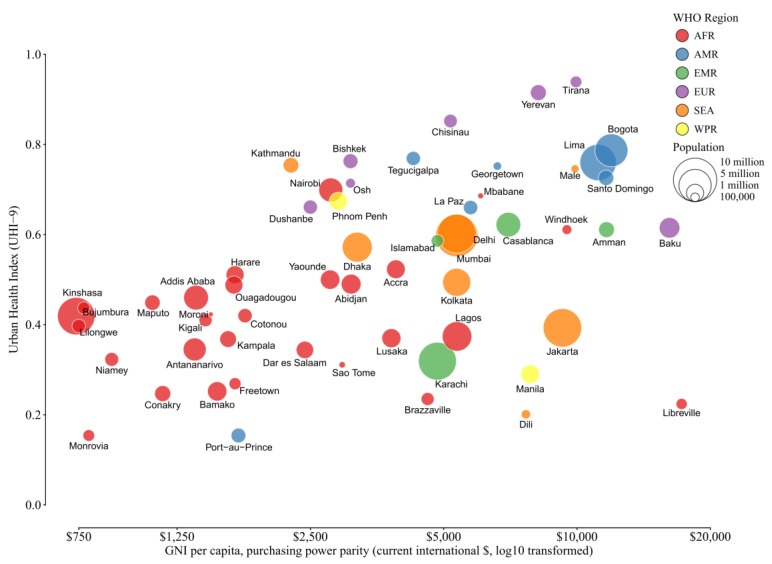
Relationship of UHI-9 and country-level per capita gross national income. The graph contains four variables: the raw UHI-9 score for the city, the log_10_ transformed per capita GNI, population size (represented by the size of the bubble), and World Health Organization (WHO) region (depicted by color).

**Figure 4:**
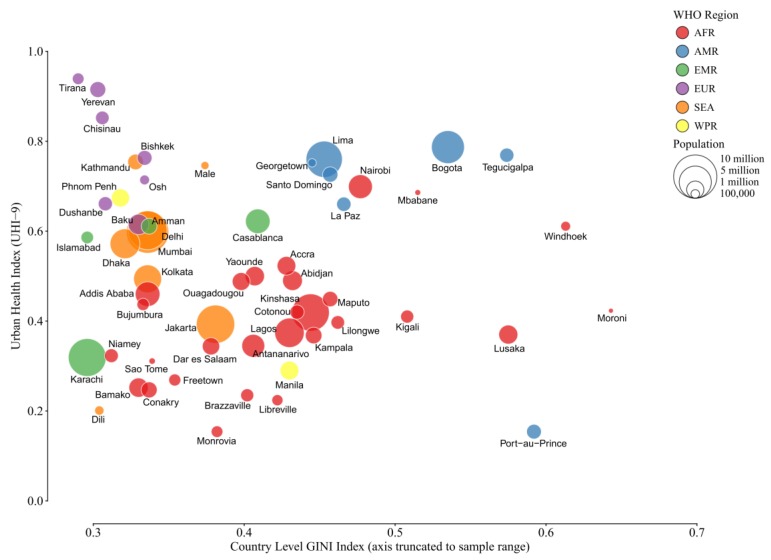
Relationship of UHI-9 and country-level index of income inequality (Gini Index). The graph contains four variables: the raw UHI-9 score for the city, country-level Gini Index, population size (represented by the size of the bubble), and WHO region (depicted by color).

Using data on UHI-9, gross national income (GNI), and the Gini Index of income inequality (Supplementary Table S3), we examined the relationship of the UHI-9 to per capita GNI (Figure 3) and level of income inequality (Figure 4). There is a clear visual relationship between level of health and per capita GNI that appears to be independent of the size of the city population (Figure 3). This relationship is dominated by the African cities, together with Port-au-Prince, Haiti, which have lower levels of health and the lowest levels of GNI. In addition, several cities have higher health levels despite being at the lower end of the income scale (Bishek and Osh, Kyrgyz Republic; Kathmandu, Nepal; Dushanbe, Tajikistan; Nairobi, Kenya; and Phnom Penh, Cambodia). In contrast, two cities have a higher GNI but a lower UHI score (Dili, Timor Leste, and Libreville, Gabon).

A trend relating the UHI-9 and the Gini Index of income inequality (Figure 4) is less evident, but several sites with disparate results are also found. For example, Bogota, Colombia, and Tegucigalpa, Honduras, have high health indices but substantial income inequality. Port-au-Prince, Haiti, stands out for its low health index and high income inequality. On this measure, the majority of African cities are clustered at the midpoint of both measurements. The comparison of city-level Gini Index with UHI-9 provides roughly the same picture with a smaller number of observations, but in general, income inequality is higher than the inequality of countries as a whole (data not shown).

### 3.3. Intracity Estimates

Using the smaller number of DHS clusters available for cities decreased the precision of estimates, but still demonstrated some important differences between the two sites we examined in detail. In Lagos, Nigeria, the disparity ratio for the 40 available clusters was 7.88 and the coefficient of variation for the UHI-9 was 0.497 (compared to the coefficient of variation for the 57 cities of 0.257). These estimates were substantially larger than those for Tegucigalpa, Honduras, whose disparity ratio was 3.27 and coefficient of variation for its 75 clusters was 0.302. The variability reported at the two sites reflected the differences in both sample size of clusters and values for individual indicators (data not shown). 

**Figure 5:**
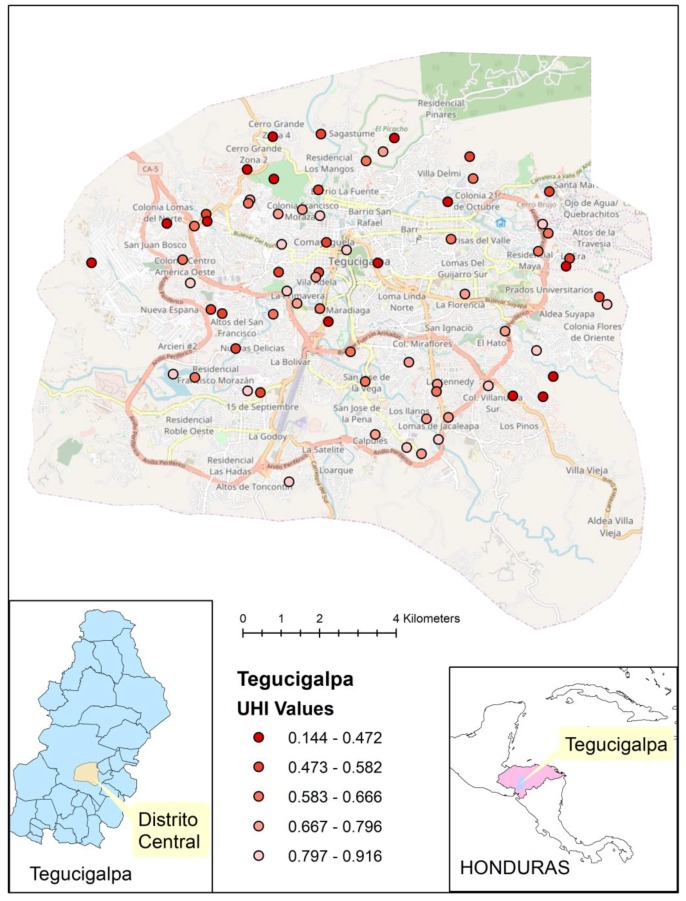
Distribution of DHS clusters and cluster UHI-9 score for Tegucigalpa, Honduras.

**Figure 6:**
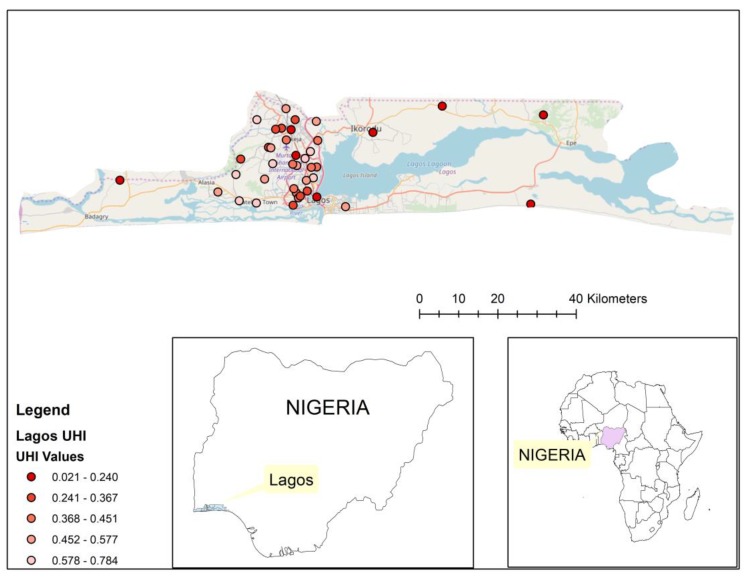
Distribution of DHS clusters and cluster UHI-9 score for Lagos, Nigeria.

With geospatial mapping, the pattern in Tegucigalpa of concentration of low health level (UHI-9 ≤0.424) in the northwest corner of the city and high health level (UHI-9 ≥0.705) in the southern portion of the city is shown in Figure 5. A similar approach in Lagos reveals a different pattern, with a concentration of low health level in the central city and higher health level at the periphery (Figure 6). The pattern in each figure is not perfectly clear, which is due, in part, to the lack of data to fill the places in between.

## 4. Discussion

The Urban Health Index is one of a number of tools that have been developed to summarize the level of health in a geographic grouping and the presence of health disparities [19,20]. Its advantages are that it does not have a fixed input, can incorporate a wide variety of data, and can be applied at various geographic scales. Its ability to be used for estimation in a small area of contiguous geographic units is an important feature, permitting public health workers to use their own data in context. An important potential disadvantage is that it is a composite statistic, and it may be difficult to sort out the effects of individual components. In this analysis, we have attempted to point to the particular role of one set of included variables, environmental indicators, that play a critical role in assessing both health level and level of inequality in geographic groupings.

Excluding environmental indicators produces a marked shift in the level and rank ordering of the cities (see Figure 2, middle panel). In this analysis, a central water supply piped into homes, improved indoor sanitation, and avoidance of indoor solid cooking fuels substantially improved the Urban Health Index for many cities and, in contrast, lowered the index for those cities that do not provide these environmental improvements. Though a direct connection between these indicators and health outcomes (overall mortality, chronic and infectious disease morbidity, childhood survival, maternal mortality) could not be shown with the data we used, this result strongly suggests a key role for municipal provision of environmental improvements.

Comparing the UHI with the GNI makes it clear that even in countries with similar income (<$2500.00 per capita), there is large variability in UHI scores. This suggests that some nations are better than others at making the best use of resources, environmental and otherwise, which is likely due to a variety of factors, including societal and political factors. Several cities were shown to have a relative high UHI despite low income levels (Figure 3), though the poorest nations (mostly in Africa) had low UHI scores and the lowest per capita incomes. At the other end of the scale, several capital cities had relatively high national incomes but were at the lower end of the UHI scale (Dili, Timor Leste, and Libreville, Gabon), suggesting difficulty in deploying resources for health. Our analysis suggests that improvements in environmental indicators may not be related to socioeconomic conditions alone. As others have found, policies and governance at the local or national level may have a stronger influence on environmental conditions and potential disease burden in cities [6,21,22,23]. The role of governance and municipal and national policy should also be an important consideration in monitoring the health of cities.

The role of income inequality is more complex (Figure 4). The distribution appears to be bimodal, in the sense that up to a Gini Index of approximately 0.5, the cities’ scattergram of the Gini Index against the UHI-9 is random. Above 0.5 there are two definable groups: higher levels of inequality may be found in cities with both higher and lower UHI scores. Taken together, these groupings suggest that income inequality may not be a determining factor of health in a large city, but distribution of available municipal resources may be more important. The economic data to explore municipal decision-making in areas of higher- and lower-income inequality would be an important area for further research.

The distribution of the UHI-9 within the two cities we examined revealed a contrasting pattern of geographic focus. The areas of poorer health were central in Lagos, Nigeria, but were at the periphery of the city in Tegucigalpa, Honduras. Such distributions have important consequences for a city’s amelioration of health disparities, since the co-distribution of health facilities, public health services, and income within a city will determine the trajectory of health levels. Although the assumption of a maldistribution of health services may be warranted, confirmatory data were not available from the sources we used, and further exploration of these multiple connections would be important to understand health inequalities.

For cities in low- and middle-income countries, our findings imply that some of the most important pathways to better health and well-being are through improvements in household level environmental conditions of the poor, a significant proportion of whom live in informal settlements. Environmental conditions in informal settlements are dire, access to clean, affordable water is still a luxury, sanitation facilities are poor or nonexistent, and living conditions threaten health and well-being. However, global monitoring platforms frequently overestimate urban water and sanitation access using metrics and monitoring strategies that have serious limitations. The Joint Monitoring Program (JMP) of the United Nations, for example, estimated in 2015 that 96% of the global population had access to improved water sources, while 82% used improved sanitation facilities [24]. Such statistics based on aggregated national averages misrepresent the scale of poor access to water and sanitation in cities and fail to account for the large proportion of the population who live in informal settlements and on the periphery of cities.

The Sustainable Development Goals (SDGs) promulgated by the United Nations in 2015 offered an ambitious path toward development: 17 goals to be attained by 2030 [16]. The SDGs provide more specific targets compared with those of the Millennium Development Goals. For example, SDG 6 is a stand-alone goal with its own targets, unlike the MDGs, which simply addressed environmental sustainability. SDG 6 emphasizes safely managed water based on three categories of increasing service provision: limited, basic, and safely managed. Safely managed water requires that drinking water should be from “an improved water source that is located on premises, available when needed and free from fecal and priority chemical contamination.” Again, local determination of the extent to which this goal is met would be critical for the health of stressed urban areas.

A central theme that emerges from these considerations is that the tools used, and others that are available, can help provide insights into health inequalities and their potential amelioration. Data on small areas can be invaluable in demonstrating the health disparities that exist within and across cities. The limiting factors are availability of data in these domains of health determinants and health outcomes, and decision-making for municipal resources. Many of these issues are best addressed on a small-area basis, but most of the data, often spotty at best, are large-scale aggregations that do not help local public health workers deal with their local problems. The use of methods developed by the Demographic and Health Surveys, the World Bank, and others for the assessment of small, contiguous geographic areas should be considered for a better understanding of how to maximize resources for health. Public health workers should consider using this approach and other tools as mechanisms for targeting funding at the city and sub-city level. Matching UHI findings to on-the-ground demonstration would be particularly effective.

## 5. Conclusions

The Urban Health Index is a flexible tool that permits local health workers to evaluate small-area configurations using local data. In this study of 57 of the world’s major cities, we demonstrated that including environmental markers (a central water supply piped into homes, improved indoor sanitation, and indoor solid fuel use) substantially changes the rank ordering of the UHI for these cities. The implication is that environmental improvements can have an important impact on health, even in the absence of changes in other markers. The availability of local data for calculating the UHI (and, by extension, other indices, markers, and determinants of health) is of central importance in amelioration of health conditions in low- and middle-income countries.

## Figures and Tables

**Table 1:** Summary statistics for the four Urban Health Indices that compare the 57 cities.

**Statistic**	**UHI-9 ^1^**	**UHI-6A ^2^**	**UHI6-B ^3^**	**UHI6-C ^4^**
Mean	0.642	0.580	0.706	0.665
Median	0.620	0.552	0.709	0.688
Minimum	0.320	0.204	0.474	0.239
Maximum	0.958	0.967	0.941	0.986
Standard deviation	0.165	0.202	0.104	0.203
Disparity ratio	2.339	3.264	1.677	2.721
Disparity gradient	0.0099	0.0122	0.0061	0.0122
Correlations *	**UHI-9**	**UHI-6A**	**UHI6-B**	**UHI6-C**
**UHI-9**	1.000			
**UHI-6A**	0.982	1.000		
**UHI-6B**	0.772	0.716	1.000	
**UHI-6C**	0.959	0.921	0.618	1.000

^1^ UHI-9: environmental, women’s health and knowledge, childhood vaccine coverage. ^2^ UHI-6A: environmental, women’s health and knowledge. ^3^ UHI-6B: women’s health and knowledge, childhood vaccine coverage. ^4^ UHI-6C: environmental, childhood vaccine coverage. * All correlation coefficients are statistically significant at *p* < 0.001.

## Supplementary Materials

The following are available online at http://www.mdpi.com/1660-4601/15/6/1216/s1: Table S1. Set of all indicators initially selected for the comparison of cities. Table S2. Description of Demographic and Health Surveys that contributed data. Table S3. Values of indicators used to calculate the UHI.

## Appendix A. Determining City Sample Selection Conditions

Appendix A contains a more detailed description of the selection of the city data. The DHS datasets include an urban/rural strata variable. However, for this project, which focused on intercity comparisons, it was necessary to identify city samples and not just the urban samples within a country. For each capital city, the DHS reports a sample size. However, the capital city is reported as an area that can contain both urban and rural areas. Therefore, an effort was made to specifically identify the urban samples within a capital city or, rather, to extract the city samples from broader urban samples. This was primarily done by cross-tabulating three variables available in the datasets: Region (v024), Type of Place of Residence (v025), and Place of Residence (v026) (Table A1). Other variables were also used, as necessary and available, to specify the urban samples of capital cities. Analysis was performed using Stata.

Table S1 in the supplementary information shows which combinations of variables were used to find the city sample sizes for each country. The focus was on capital cities; in some cases, additional major cities were also identified and included in the analysis when possible (e.g., Delhi, Kolkata, and Mumbai in India). While the best attempt was made to uniformly extract the urban city samples across all the countries, this was not possible, as the country datasets varied in terms of the availability and coding of the place-related variables.

**Table A1:** Variable codes in the DHS used to identify the urban sample from a capital city in each sample dataset.

Sample	Region	Type of Place of Residence (Urban/Rural)	(De Facto) Place of Residence (Capital/Small City/Town/Countryside)
Household	hv024	hv025	hv026
Individual (female)	v024	v025	v026
Men	mv024	mv025	mv026
Children	v024	v025	v026
