# Supplementary material for: Measuring the Impact of Environment on the Health of Large Cities
Source: Int J Environ Res Public Health. 2018 Jun 9;15(6):1216. doi: 10.3390/ijerph15061216 (PMC6025373; doi:10.3390/ijerph15061216)
Supplement: Supplementary file 1 [file ijerph-15-01216-s001.pdf]

Table S1. Set of all indicators initially selected for the comparison of cities.

**Domain**	**Indicator**	**DHS file to use for calculation**	**Variable (DHS VI)**
Environment	Proportion of households with piped water into dwelling or yard/plot (on the premises)	Household Recode (HR)	hv201/hv202
Environment	Proportion of households with access to improved sanitation as defined by WHO/UNICEF JMP	Household Recode (HR)	hv205
Environment	Proportion of households that are not using solid fuel for cooking in the home	Household Recode (HR)	hv226
Environment	Proportion of households that do not have environmental tobacco smoke in the home ever	Household Recode	hv252
Chronic disease risk factors (Smoking (male))	The percentage of males (15-49) who do not smoke cigarettes	Male Recode (MR)	mv463a
Education	Proportion of men (15-49) who can read all of a phrase on a card	Male Recode (MR)	mv155
Education	Proportion of men (15-49) who have completed secondary education	Male Recode (MR)	mv149
Chronic disease risk factors (Smoking (female))	The percentage of females (15-49) who do not smoke cigarettes	Individual Recode	v463a
Infectious disease risk factors (Knowledge of HIV)	The percent of respondents (women 15-49) who, in response to a prompted question, say that people can protect themselves from contracting HIV by having sex only with one faithful, uninfected partner	Individual Recode (IR)	v754dp
Infectious disease risk factors (Knowledge of HIV)	The percent of respondents (women 15-49) who, in response to a prompted question, say that people can protect themselves from contracting HIV by using condoms	Individual Recode (IR)	v754cp
Education	Proportion of women (15-49) who can read all of a phrase on a card	Individual Recode (IR)	v155
Education	Proportion of women (15-49) who have completed secondary education	Individual Recode (IR)	v149
Chronic disease risk factor (Body mass index)	Proportion of non-pregnant women (15-49) who have a BMI < 30	Individual Recode	v213, v445, v447
Child health and health coverage	Proportion of children (1-4 years of age) who have received all three doses of the DPT vaccine.	Child Recode (KR)	b8, h7
Child health and health coverage	Proportion of children (1-4 years of age) who have received all three doses of the polio vaccine.	Child Recode (KR)	b8, h8
Child health and health coverage	Proportion of children (1-4 years of age) who have received the measles vaccine.	Child Recode (KR)	b8, h9

Table S2: Description of Demographic Health Surveys that contributed data

Country	Year	Country Code	Selected City	Household Sample Size	Women Sample Size	Men Sample Size	Children Sample Size	Variables Used to Identify City Sample
Albania	2008	AL	**Tirana**	1366	1142	520	177	V024=4 & V025=1
Armenia	2010	AM	**Yerevan**	1149	987	283	176	V024=11 & V025=1
Azerbaijan	2006	AZ	**Baku**	1166	1312	422	201	V024=1 & V025=1
Bangladesh	2011	BD	**Dhaka**	1322	1345	138	177	v024=3 & v025=1 & V023=3
Benin	2011-12	BJ	**Cotonou**	1913	1949	508	861	V024=8 & V025=1
Bolivia	2008	BO	**La Paz**	2513	2315	798	694	v024=2 & v025=1
Burkina Faso	2010	BF	**Ouagadougou**	939	1333	624	480	V024=3 & V025=1
Burundi	2010	BU	**Bujumbura**	912	1212	722	563	V024=1 & V025=1
Cambodia	2010	KH	**Phnom Penh**	799	1251	534	302	v024=8 & v025=1
Cameroon	2011	CM	**Yaounde**	1156	1352	570	573	V026=1 & V025=1 & V024=12
Chad	2004	TD	**N'Djamena**	1169	1324	549	900	V024=9 & V025=1
Colombia	2010	CO	**Bogota**	3604	3690	x	781	v024=5 & v025=1
Comoros	2012	KM	**Moroni**	770	865	394	276	V023=7 & V025=1
Congo (Brazzaville)	2011-12	CG	**Brazzaville**	1173	1180	584	580	V024=11 & V025=1
Congo Democratic Republic	2013	CD	**Kinshasa**	1214	1804	762	806	V024=1 & V025=1
Cote d'Ivoire	2011-12	CI	**Abidjan**	1137	1400	641	515	V024=11& V025=1
Dominican Republic	2013	DR	**Santo Domingo**	434	343	343	103	sprovin=1
Ethiopia	2011	ET	**Addis Ababa**	1524	1741	1237	315	V024=14 & V025=1

Country	Year	Country Code	Selected City	Household Sample Size	Women Sample Size	Men Sample Size	Children Sample Size	Variables Used to Identify City Sample
Gabon	2012	GA	**Libreville**	1562	1557	900	650	V024=1 & V025=1
Ghana	2008	GH	**Accra**	1481	622	528	189	V024=3 & V025=1
Guinea	2012	GN	**Conakry**	1010	1465	620	609	V024=2 &V025=1 & V026=0
Guyana	2009	GY	**Georgetown**	598	554	394	116	V024=4 & V026=0 & V025=1
Haiti	2012	HT	**Port-au-Prince**	1761	2361	1365	665	v026=0
Honduras	2011-12	HN	**Tegucigalpa**	1276	1351	345	335	hv022=16
India	2005	IA	**New Delhi**	3109	3105	1336	935	v024=7& v025=1 & V026=0
India	2005	IA	**Kolkata**	2337	2511	1065	503	v024=19 & v025=1 & V026=0
India	2005	IA	**Mumbai**	4928	5332	5301	1287	v024=27 & v025=1 & V026=0
Indonesia	2012	ID	**Jakarta**	2118	2391	466	614	v024==31 & v025==1
Jordan	2012	JO	**Amman**	1553	1106	x	661	V023=11 & V025=1
Kenya	2008-09	KE	**Nairobi**	1108	952	399	322	V024=1 & V025=1 & V026=0
Kyrgyz Republic	2012	KY	**Bishkek**	918	983	234	268	V024=8 & V025=1
Kyrgyz Republic	2012	KY	**Osh**	737	641	157	198	V024=9 & V025=1

Country	Year	Country Code	Selected City	Household Sample Size	Women Sample Size	Men Sample Size	Children Sample Size	Variables Used to Identify City Sample
Liberia	2013	LB	**Monrovia**	1062	1293	505	512	hv023=21
Madagascar	2008	MD	**Antananarivo**	1507	1653	741	547	V024=11 & V025=1
Malawi	2010	MW	**Lilongwe**	432	480	157	243	v026==0
Maldives	2009	MV	**Male**	944	1041	274	371	V024=1
Mali	2012-13	ML	**Bamako**	1579	1970	811	1178	V024=9 & V025=1
Moldova	2005	MB	**Chisinau**	2922	2031	623	285	V024=4 & V025=1
Morocco	2003-2004	MA	**Casablanca**	1290	1837	x	370	V024=9 & V025=1 & v026=0
Mozambique	2011	MZ	**Maputo**	1297	1724	432	679	V024=11 & V025=1
Namibia	2013	NM	**Windhoek**	888	1005	423	305	V024=6 & V025=1
Nepal	2011	NP	**Kathmandu**	509	539	232	110	V023=5 & V025=1
Niger	2012	NI	**Niamey**	1135	1379	562	799	V024=8 & V025=1 & V026=0
Nigeria	2013	NG	**Lagos**	1729	1482	701	753	HV022=71 & v025=1
Pakistan	2012	PK	**Karachi**	1290	1184	308	620	v024=2 & v025=1 & V026=0

Country	Year	Country Code	Selected City	Household Sample Size	Women Sample Size	Men Sample Size	Children Sample Size	Variables Used to Identify City Sample

Pakistan	2012	PK	**Islamabad**	828	627	193	361	v024=6 & v025=1 & V026=0
Peru	2012	PE	**Lima**	2179	2176	x	492	v024=15 & v025=1 & V026=0
Philippines	2013	PH	**Manila**	1683	2249	x	638	v024=1 & v025=1
Rwanda	2010	RW	**Kigali**	1269	1618	785	648	V024=1 & V025=1
Sao Tome and Principe	2008-2009	ST	**Sao Tome**	699	635	440	301	V024 =1 & V025=1
Senegal	2012-13	SN	**Dakar**	395	549	x	749	V024=1 & V025=1
Sierra Leone	2013	SL	**Freetown**	1584	2337	1069?	892	V024=4 & V025=1
Swaziland	2006-2007	SZ	**Mbabane**	426	356	308	127	V024=1 & V026=0 & V025=1
Tajikistan	2012	TJ	**Dushanbe**	1422	1733	x		V024=1 & V025=1
Tanzania	2010	TZ	**Dar es Salaam**	352	410	100	158	V024=7 & V025=1 & V026=0
Timor-Leste	2009-10	TL	**Dili**	880	1047	351	580	v024=6 & v025=1
Uganda	2011	UG	**Kampala**	1062	1039	238	474	V024=1 & V025=1
Ukraine	2007	UA	**Kyiv**	728	339	180	55	V024=1 & V026=0
Zambia	2007	ZM	**Lusaka**	527	614	583	302	V024=5 & V026=0 & V025 = 1
Zimbabwe	2010-11	ZW	**Harare**	1121	1196	894	445	V024=9 & V025=1

Table S3: Values of indicators used to calculate the UHI

Country	City	% piped onto premises	% improved sanitation	% not using solid fuels for cooking in the home	% women who completed secondary or higher	% women who believe condom prevents HIV	% women who believe having one partner prevents HIV	% with dpt3 age 1-4	% with polio 3 age 1-4	% with measles vaccine age 1-4
**Albania**	Tirana	94.80	98.98	97.58	70.67	90.63	92.95	100.00	100.00	99.44
**Armenia**	Yerevan	99.13	98.26	100.00	94.43	84.50	91.17	94.15	93.57	94.12
**Azerbaijan**	Baku	90.21	88.45	99.92	53.20	66.25	49.62	86.67	82.35	89.76
**Bangladesh**	Dhaka	60.59	33.51	75.93	36.41	70.63	77.35	94.67	94.08	89.94
**Benin**	Cotonou	66.44	35.65	28.54	14.57	82.71	91.36	75.67	50.06	87.29
**Bolivia**	La Paz	95.90	64.09	98.53	53.61	80.29	78.78	80.47	80.93	78.65
**Burkina Faso**	Ouagadougou	48.35	56.98	37.38	8.40	80.48	88.67	87.95	88.13	92.27
**Burundi**	Bujumbura	56.14	33.99	10.99	12.95	93.48	88.64	92.32	85.58	96.81
**Cambodia**	Phnom Penh	83.35	89.99	75.91	28.22	67.04	94.23	91.64	93.00	90.67
**Cameroon**	Yaoundé	39.97	42.47	77.99	22.41	80.69	85.83	78.57	64.82	84.27
**Chad**	N'Djamena	53.94			7.10	52.35	72.12	50.40	47.82	57.62
**Colombia**	Bogotá	99.86	90.21	99.97	60.22	82.06	75.11	94.05	75.43	92.41
**Comoros**	Moroni	41.56	24.03	82.08	28.21	79.51	84.46	71.26	69.35	76.15
**Congo**	Brazzaville	43.99	13.64	42.56	18.47	77.75	84.23	61.29	47.75	86.60
**Congo Democratic Republic**	Kinshasa	48.93	19.69	24.77	43.46	69.19	85.41	84.54	72.46	93.96
**Cote dÍvoire**	Abidjan	87.32	40.99	71.16	14.36	74.22	79.08	78.15	72.67	82.31
**Dominican Republic**	Santo Domingo	94.83	82.49	98.85	52.77	83.09	90.38	81.71	58.59	91.03
**Ethiopia**	Addis Ababa	72.24	18.70	45.41	28.20	82.61	65.65	88.63	82.72	94.00
**Gabon**	Libreville	82.22	40.85	99.42	16.89	85.03	89.18	47.39	30.05	80.20
**Ghana**	Accra	44.50	25.93	48.95	34.08	77.15	89.77	89.20	84.83	96.07
**Guinea**	Conakry	56.44	34.36	6.44	15.36	76.65	84.07	52.65	50.09	82.12
**Guyana**	Georgetown	87.12	92.98	100.00	51.81	94.03	93.84	84.55	63.64	84.55

**Haiti**	Port-au-Prince	6.94	39.92	16.64	14.99	87.10	94.10	63.40	55.08	67.01
**Honduras**	Tegucigalpa	93.42	72.65	93.57	40.61	77.67	93.10	94.14	95.05	93.60
**India-Delhi**	Delhi	70.34	54.91	89.58	33.17	83.14	89.69	74.68	82.08	80.42
**India-Kolkata**	Kolkata	46.38	41.85	84.04	24.05	66.10	75.11	82.73	86.08	84.02
**India - Mumbai**	Mumbai	81.29	51.44	88.86	28.24	73.33	80.46	79.03	81.95	88.74
**Indonesia**	Jakarta	14.73	80.17	99.57	57.01	53.33	68.68	77.03	79.00	84.89
**Jordan**	Amman	51.86	99.71	100.00	44.22	55.57	75.74	99.38	99.38	97.82
**Kenya**	Nairobi	82.49	50.00	87.07	58.19	86.85	96.50	83.50	59.60	95.39
**Kyrgyz Republic - Bishek**	Bishkek	98.04	90.52	99.67	89.32	78.96	88.88	73.54	59.62	95.38
**Kyrgyz Republic-Osh**	Osh	89.82	98.91	98.24	76.44	58.17	75.04	96.24	88.72	97.42
**Liberia**	Monrovia	3.20	27.68	4.33	22.51	83.49	86.51	81.34	70.34	84.60
**Madagascar**	Antananarivo	27.80	19.58	9.10	24.56	88.44	92.52	87.79	78.57	89.15
**Malawi**	Lilongwe	44.21	23.84	13.69	27.50	69.67	86.19	91.00	73.93	97.62
**Maldives**	Male	92.05	97.03	100.00	15.85	84.68	95.56	97.77	92.82	94.75
**Mali**	Bamako	32.68	40.22	5.45	9.04	76.60	82.33	72.96	50.63	84.34
**Moldova**	Chisinau	89.14	88.81	99.86	56.08	86.45	88.04	93.17	96.40	97.36
**Morocco**	Casablanca	85.27	86.59	99.69	14.40	60.51	93.29	97.49	97.48	94.96
**Mozambique**	Maputo	64.69	31.61	33.33	14.04	73.29	80.26	86.18	73.23	94.48
**Namibia**	Windhoek	67.34	51.13	94.81	51.24	91.42	92.63	65.69	58.36	87.50
**Nepal**	Kathmandu	57.76	53.24	91.55	51.39	92.44	93.19	95.19	97.12	93.27
**Niger**	Niamey	51.19	30.57	12.00	7.40	69.84	85.46	82.37	80.74	83.56
**Nigeria**	Lagos	4.86	27.99	94.22	65.05	77.25	88.65	80.44	61.77	80.95
**Pakistan-Karachi**	Karachi	75.89	98.06	99.46	51.77	51.96	81.47	63.15	73.31	74.56
**Pakistan-Islamabad**	Islamabad	21.89	93.36	92.36	64.91	65.70	84.42	83.43	77.52	86.34
**Peru**	Lima	91.79	83.75	99.22	73.94	79.96	88.12	82.04	84.93	83.40
**Philippines**	Manila	40.46	67.62	87.27	79.01	55.49	36.30	90.69	89.87	92.10
**Rwanada**	Kigali	36.33	41.92	5.69	20.33	94.50	92.95	98.53	98.05	97.88
**Sao Tome and Principe**	São Tomé	47.07	42.35	49.21	2.52	78.64	83.73	88.36	78.70	85.20
**Senegal**	Dakar	82.23	43.07	74.76	8.20			87.18	70.83	89.44

**Sierra Leone**	Freetown	19.79	29.10	4.75	23.87	86.08	90.66	72.94	71.43	85.73
**Swaziland**	Mbabane	67.14	51.17	69.25	43.54	95.77	96.35	93.04	63.79	94.83
**Taijikistan**	Dushanbe	96.90	93.46	99.44	62.61	57.52	66.43	92.86	88.26	95.17
**Tanzania**	Dar es Salaam	12.50	19.60	24.22	9.27	80.84	93.90	100.00	96.38	98.54
**Timor-Leste**	Dili	49.89	73.18	26.93	46.70	61.37	78.08	62.50	26.22	71.53
**Uganda**	Kampala	33.15	19.30	21.23	26.76	87.56	91.71	76.23	63.53	85.75
**Ukraine**	Kyiv	99.59	98.90	100.00	83.48	91.77	88.11			
**Zambia**	Lusaka	29.60	26.00	48.96	21.17	69.33	88.27	58.46	76.25	88.85
**Zimbabwe**	Harare	67.35	37.02	89.29	11.12	80.99	92.09	79.30	81.55	84.79
